# Traumatic Diaphragmatic Rupture With Pericardial Tear and Transdiaphragmatic Herniation of the Stomach

**DOI:** 10.7759/cureus.29473

**Published:** 2022-09-22

**Authors:** Naveen Naik Mude, Sagar Prakash, Oseen Shaikh, Chellappa Vijayakumar, Uday Kumbhar

**Affiliations:** 1 Surgery, Jawaharlal Institute of Postgraduate Medical Education and Research, Puducherry, IND

**Keywords:** laparotomy, diaphragmatic rupture, blunt abdominal trauma, penetrating abdominal trauma, pericardial tear

## Abstract

Traumatic diaphragmatic rupture is uncommon in blunt or penetrating abdominal trauma. Diaphragmatic injury associated with pericardial tear is even rarer. Here, we report a case of a 23-year-old female who presented with complaints of chest pain, abdomen pain, and breathlessness following blunt trauma abdomen. An imaging study showed the presence of transdiaphragmatic herniation of the stomach inside the thorax. Emergency exploratory laparotomy was done, and we found a large diaphragmatic defect with a pericardial tear with herniation of the stomach. Both diaphragmatic and pericardial tears were repaired primarily. Postoperatively, the patient improved well without any complications.

## Introduction

In trauma patients, thoracic injuries are the third most common, with an overall mortality rate of about 10% [1]. Traumatic diaphragmatic rupture is an uncommon injury resulting from penetrating injury to the chest or abdomen or from high-velocity blunt trauma to the abdomen, and challenging to diagnose as it is a rare pathology and can be a precedent to severe complications if left untreated [1]. Detecting the injury is difficult, as the associated symptoms are often non-specific. Of all trauma cases, it presents in less than 0.5% of cases, with the majority caused by a penetrating mechanism, followed by blunt injuries [2-4]. Pericardial or cardiac injuries are the most lethal in the setting of penetrating thoracic trauma. Most patients who sustained these injuries were found dead at the scene. Chest x-ray and contrast-enhanced computed tomography (CT) are used to confirm the diagnosis. Some patients may have indistinct imaging features. A high degree of clinical suspicion might be required in hemodynamically stable patients. Some patients may subsequently become unstable. Immediate exploration and reduction of hernia and repair of the diaphragmatic and pericardial tear with tube thoracostomy is the treatment with supportive care.

## Case presentation

A 23-year-old female presented to our emergency room with a history of road traffic accident five hours back. The patient had complaints of breathlessness, chest pain, and abdominal pain. On examination, the Glasgow coma scale (GCS) was 15, pulse rate was 108 beats per minute, and blood pressure of 106/60 mm Hg. There were decreased breath sounds noted on the left side of the chest. There were a few abrasions over the left side of the abdomen and thorax. Per abdomen was soft, with minimal tenderness in the epigastric region and left hypochondriac region. The pelvis compression test was negative. However, the patient had tenderness at the right sacroiliac joint. 

**Figure 1:**
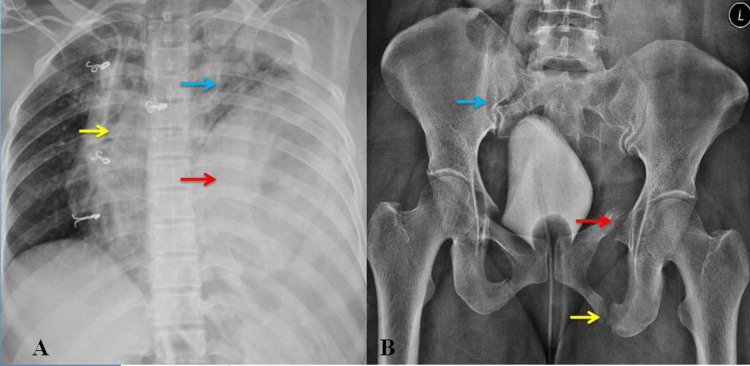
(A) Chest x-ray showing a shift of the mediastinum towards the right (yellow arrow), the collapse of the left lung (blue arrow), and herniated stomach in the thorax (red arrow); (B) Pelvis x-ray showing fractures of the right sacroiliac joint (blue arrow), left superior pubic ramus (red arrow), and inferior pubic ramus (yellow arrow).

After the initial survey and stabilization, the patient was taken for radiological imaging studies. A plain x-ray showed the haziness of the left thoracic cavity and the elevation of the diaphragm with the collapse of the left lung. There was the presence of a suspicious gastric shadow in the left hemithorax with the mediastinal shift towards the right. The nasogastric tube was seen above the hemidiaphragm. X-ray of the pelvis showed a fracture at the right sacroiliac joint, fracture of the left superior pubic ramus, and fracture of the left inferior pubic ramus (Figure 1).

**Figure 2:**
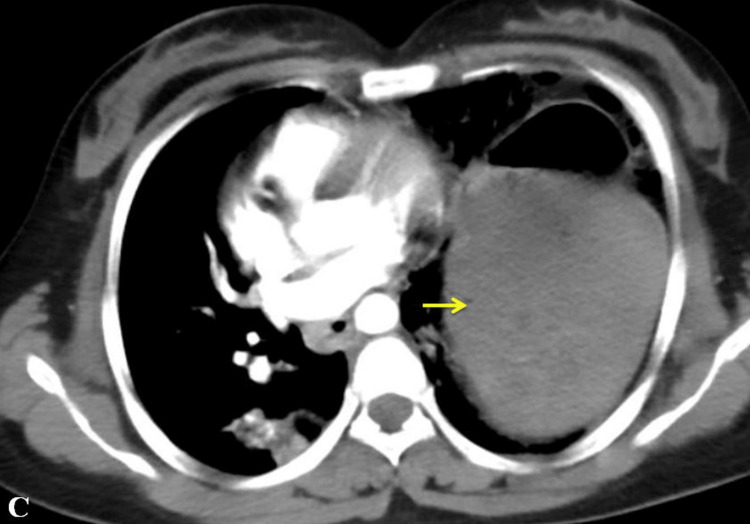
CT thorax showing herniated stomach in the left hemithorax (arrow) air-fluid level.

The patient underwent extended focussed assessment with sonography in trauma (e-FAST) and was found to have minimal hemoperitoneum and left hemothorax with grade I splenic hematoma. CT thorax and abdomen were done as the patient was hemodynamically stable, which showed herniation of the stomach through the left hemidiaphragm (posteromedial) with an air column at the epicardial fat pocket. There was also evidence of left hemothorax, grade I splenic hematoma with mild hemoperitoneum. There was no clear evidence of pericardial laceration (Figure 2).

**Figure 3:**
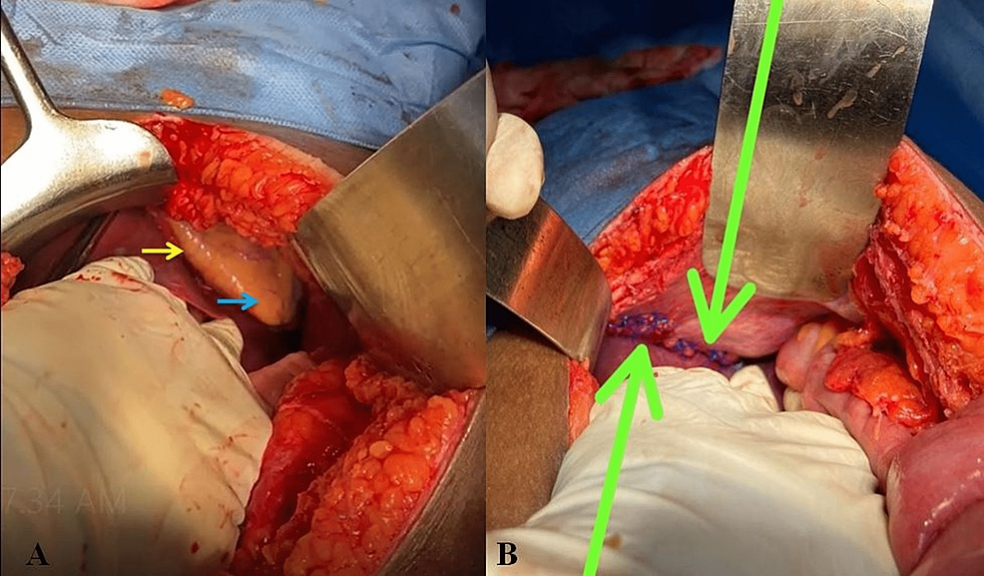
Intraoperative images showing (A) diaphragmatic rent (yellow arrow), pericardial fat (blue arrow), and (B) sutured diaphragmatic rent (arrows).

The patient underwent an emergency exploratory laparotomy. Intraoperatively, we found minimal hemoperitoneum with a gross defect of the left hemidiaphragm and a pericardial laceration. There was herniation of the stomach through the diaphragmatic rent. There was no other organ injury or bowel injury except for the subcapsular hematoma of the spleen. The stomach was reduced into the abdomen, and the pericardial defect was closed with a simple interrupted polypropylene suture. The diaphragm was closed primarily without tension, and the left side tube thoracostomy was done (Figure 3).

Postoperatively, the patient improved without any complications and was discharged after one week.

## Discussion

Traumatic diaphragmatic hernia is a rare pathology that is challenging to diagnose. The most common cause is motor vehicle collisions. Of all trauma cases, it presents in less than 0.5% of cases, with the majority caused by a penetrating mechanism, followed by blunt injuries [4]. One study reported that 40%, 25%, and 5% of patients had blunt diaphragmatic injuries with pelvic fracture, spleen and liver injuries, and thoracic aortic injuries, respectively [5]. Only a few cases of isolated rupture of the pericardium or combined diaphragmatic and pericardial ruptures have been reported [6]. Also, few cases of pericardial tear with intra-pericardial diaphragmatic hernia have been reported [7]. Pericardial or cardiac injuries are the most lethal in the setting of penetrating thoracic trauma [4].

The posterolateral aspect of the hemithorax is the most common site of rupture of the diaphragm because of its origin from the pleura-peritoneal membrane, which is structurally weak. The stomach, followed by the spleen, is the most common organ to herniate. In blunt trauma, the left pleuro-pericardium is the most common site of pericardial injury. The left and right ventricles are injured approximately 40% of the time. The right atrium is injured 24% of the time, and the left atrium is injured 3% of the time [4]. In our patient, there was a laceration of the diaphragm at the posteromedial aspect and a pericardial laceration at the left pleuro-pericardial region with an extension over the diaphragmatic surface of the pericardium. There was no evidence of atrial or ventricular damage.

The most accepted pathophysiology of this type of injury is that following blunt trauma to the abdomen, increased intra-abdominal pressure will create a sufficiently high-pressure gradient between the abdomen and chest to cause rupture and subsequent intrathoracic herniation of visceral contents. Typically, a positive pressure gradient of 7-20 cm of H_2_O exists between the intraperitoneal and intrapleural pressures. However, this gradient can exceed 100 cm of H_2_O during blunt trauma, leading to rupture and herniation [4]. We hypothesized that the pericardial tear was probably due to diaphragmatic rupture as a result of a high-impact injury to the abdomen. This might have been due to adherence of the pericardium to the diaphragm.

The most common presentations include chest pain, breathlessness, and abdominal distention. Few patients may have reduced breath sounds on the affected side. This can lead to misdiagnosis as pneumothorax. This could lead to improper interventions such as intercostal chest tube insertion through a herniated organ. Due to herniation of bowel loops into the thorax, there may be symptoms relating to cardiopulmonary dysfunction, perforation, strangulation, or intestinal obstruction.

Tiny lacerations, often tamponade, fibrous pericardium, can seal off with blood and clot accumulating within the pericardial space leading to hemopericardium [4]. Few patients may not develop any consequences, in whom only an isolated pericardial laceration is present. The patient can have two more consequences if the pericardial laceration is large. The heart may get dislocated, or there can be torsion. If the torsion of the heart occurs, it will be along the axis made by the inferior vena cava and the great vessels. Our patient presented with pain abdomen, breathlessness, and chest pain. The patient had decreased breath sounds on the left side of the chest; however, there were no apparent signs of diaphragmatic and pericardial laceration.

It may be challenging to diagnose diaphragmatic injuries despite a thorough history, physical examination, and recent advances in imaging modalities. Hence, imaging modalities play a crucial role in diagnosing, triaging, and managing trauma patients, including e-FAST or echocardiography, conventional radiography, and CT. The yield of chest x-rays diagnosing diaphragmatic ruptures is low, leading to a missed or delayed diagnosis as features are often masked by associated pleural effusion, haemothorax, lung contusion, emphysema, pneumothorax, atelectasis, and non-specific elevation of the diaphragm. However, it has a limited role in identifying pericardial and cardiac injuries. The findings on a plain chest radiograph in a pericardial injury include alteration of the cardiac axis, unusual cardiac silhouette contour, or an enlarged cardiac outline (secondary to a pericardial effusion), and pneumopericardium.

The axial or coronal CT images can aid differentiation if the plain radiography findings are challenging to interpret. CT is vital in assessing stable polytrauma patients, especially in patients with pericardial and cardiac injuries and other trauma-associated injuries. The sensitivity for left and right-sided diaphragmatic injuries include 78% and 50%, respectively, whereas specificity is 100% for left-sided diaphragmatic injuries and 83% for right-sided ones [4]. The collar sign or hourglass sign may be seen in a diaphragmatic hernia, seen on a coronal section of CT or magnetic resonance imaging (MRI) and barium studies, which is the waist-like or collar-like appearance of herniated organs through the defect in the diaphragm. The position of the nasogastric tube in the stomach in a supradiaphragmatic location is another sign of a diaphragmatic hernia. The CT images of a large pericardial tear that causes cardiac herniation include alteration of the normal cardiac axis, alteration of the normal cardiac contour (collar sign), empty pericardial sac sign, and displacement of the heart. Conversely, this may also result in cardiac herniation into the abdominal cavity. On the other hand, there may also be herniation of the bowel with bowel gas noted within the pericardial sac following injury to the diaphragmatic surface of the pericardium [8].

Historically, treating diaphragmatic rupture from a blunt mechanism includes surgical reduction and diaphragm repair through an abdominal laparotomy approach, allowing the surgeon to simultaneously treat the associated intra-abdominal injuries [4]. Prosthetic closure of pericardial defects can be tried when there is a significant defect with tissue loss where tissue approximation is impossible. If necessary, sutures are passed around the rib and supported by a Teflon pledget [5]. The ruptured diaphragm is repaired by approximation of rent by applying simple sutures. For large rents, a mesh placement can be done. Laparoscopic repair is becoming popular. Minimally invasive procedures through the thoracic and abdominal approach are becoming popular as repair is technically easier in experienced hands. Our patient underwent laparotomy and primary repair of the pericardial and diaphragmatic laceration.

## Conclusions

Concomitant diaphragmatic and pericardial rupture following blunt trauma to the abdomen is very rare. Few patients may have associated cardiac injuries. Pericardial lacerations may be challenging in routine imaging studies like CT scans. Very few patients may have clear evidence of pericardial tear in the CT scan. Emergent exploration has to be done to prevent the development of complications. Primary repair of the pericardial tear and diaphragmatic laceration can be done.

## References

[1] Kumar S, Pol M, Mishra B, Sagar S, Singhal M, Misra MC, Gupta A (2015). Traumatic diaphragmatic injury: a marker of serious injury challenging trauma surgeons. Indian J Surg.

[2] Fair KA, Gordon NT, Barbosa RR, Rowell SE, Watters JM, Schreiber MA (2015). Traumatic diaphragmatic injury in the American College of Surgeons National Trauma Data Bank: a new examination of a rare diagnosis. Am J Surg.

[3] Bhatti UH, Dawani S (2015). Large bowel obstruction complicating a posttraumatic diaphragmatic hernia. Singapore Med J.

[4] Shaban Y, Elkbuli A, McKenney M, Boneva D (2020). Traumatic diaphragmatic rupture with transthoracic organ herniation: a case report and review of literature. Am J Case Rep.

[5] Özalp T, Küpeli M, Sönmezoğlu Y, Çakmak A, Akgül S, Fazlioğlu M, Tokat C (2017). Blunt diaphragmatic injuries: pericardial ruptures. Indian J Surg.

[6] Gao R, Jia D, Zhao H, WeiWei Z, Yangming WF (2018). A diaphragmatic hernia and pericardial rupture caused by blunt injury of the chest: a case review. J Trauma Nurs.

[7] Garcia LD, de Melo AS, Cañete LA (2018). Traumatic rupture of the diaphragm with pericardial diaphragmatic hernia. Radiol Bras.

[8] Sherren PB, Galloway R, Healy M (2009). Blunt traumatic pericardial rupture and cardiac herniation with a penetrating twist: two case reports. Scand J Trauma Resusc Emerg Med.

